# Dietary antioxidants and liver enzymes in Rafsanjan, a Region in Southeast Iran

**DOI:** 10.1038/s41598-023-35385-0

**Published:** 2023-05-26

**Authors:** Morteza Khademalhosseini, Elham Ranjbar, Rezvan Mohammadi, Parvin Khalili, Mahya Mehran, Nazanin Jalali, Zohreh Rajabi, Zahra Jamali

**Affiliations:** 1 Clinical Research Development Unit (CRDU), Ali-Ibn Abi-Talib Hospital, Rafsanjan University of Medical Sciences, Rafsanjan, Iran; 2 Department of Pathology, Medical School, Rafsanjan University of Medical Sciences, Rafsanjan, Iran; 3 Immunology of Infectious Diseases Research Center, Rafsanjan University of Medical Sciences, Rafsanjan, Iran; 4 Department of Medical Biotechnology, School of Advanced Technologies in Medicine, Shahid Beheshti University of Medical Sciences, Tehran, Iran; 5 Student Research Committee, Department of Medical Biotechnology, School of Advanced Technologies in Medicine, Shahid Beheshti University of Medical Sciences, Tehran, Iran; 6 Social Determinants of Health Research Center, Rafsanjan University of Medical Sciences, Rafsanjan, Iran; 7 Department of Epidemiology, School of Public Health, Rafsanjan University of Medical Sciences, Rafsanjan, Iran; 8 Non-Communicable Diseases Research Center, Rafsanjan University of Medical Sciences, Rafsanjan, Iran; 9 Neurology Department, School of Medicine, Rafsanjan University of Medical Sciences, Rafsanjan, Iran; 10 Clinical Research Development Unit (CRDU), Moradi Hospital, Rafsanjan University of Medical Sciences, Rafsanjan, Iran; 11 Clinical Research Development Unit (CRDU), Niknafs Hospital, Rafsanjan University of Medical Sciences, Rafsanjan, Iran

**Keywords:** Diseases, Gastroenterology

## Abstract

Oxidative stress has been considered the main contributor to liver injury. Dietary antioxidants would be expected to improve liver function. The hepatoprotective effects of antioxidants are controversial. In the present study, the associations of some dietary antioxidants and the levels of serum liver enzymes were examined. This cross-sectional study was conducted using the Rafsanjan Cohort Study (RCS) data as a population-based prospective cohort which is a part of the Prospective Epidemiological Research Studies in IrAN (PERSIAN). A total of 9942 participants aged 35–70 years old were included in this study. Among this population, 4631 (46.59%) were male, and 5311 (53.42%) were female. Dietary intakes were collected by a validated food frequency questionnaire (FFQ) with 128 items. Aspartate transaminase (AST), Alanine transaminase (ALT), γ-glutamyl transferase (GGT), and Alkaline phosphatase (ALP) were measured by a biotecnica analyzer. Dichotomous logistics regression models were used to investigate the association between the elevated liver enzymes and intake of dietary antioxidants using crude and adjusted models. In the adjusted model, in subjects with higher consumption of Se, Vit A, Vit E, β-carotene, α-carotene, and β-cryptoxanthin, the odds ratios of elevated ALP were decreased compared to the reference group (ORs 0.79 (0.64–0.96), 0.80 (0.66–0.98), 0.73 (0.60–0.89), 0.79 (0.64–0.96), 0.78 (0.64–0.95), 0.80 (0.66–0.98), and 0.79 (0.64–0.98), respectively). Subjects with higher consumption of Se, Vit A, Vit E, and provitamin A carotenoids (β-carotene, α-carotene, β-cryptoxanthin) showed decreased odds of elevated ALP. These findings support the hypothesis that Se, Vit A, Vit E, and provitamin A carotenoids may be associated with improvements in ALP and act as suppressors against the development of liver injury.

## Introduction

As one of the most important organs in the body, the liver plays a key role in carbohydrate, protein, and fat metabolism^1^. Moreover, this organ is responsible for many of the body's metabolic functions, including the breakdown of toxic and waste materials and the removal of harmful substances from the body^2^. The liver plays a vital role in creating balance in the body, regulating the substances necessary for supplying energy, and feeding the tissues^3^. The liver contains high amounts of enzymes, such as cytoplasmic enzymes Aspartate transaminase (AST), Alanine transaminase (ALT), γ-glutamyl transferase (GGT), and Alkaline phosphatase (ALP), which are considered as the most important functional indicators of liver health^4^. High levels of liver enzymes are related to some diseases, including hypertension^5^, diabetes^6^, and non-alcoholic fatty liver disease (NAFLD)^7^.

Oxidative stress refers to an imbalance in the prooxidant-antioxidant system, an imbalance towards oxidants that lead to tissue damage^8^. Under normal conditions, the aerobic metabolism of the liver produces constant amounts of prooxidants, such as reactive oxygen species (ROS), which are balanced by the consumption of these substances at the same rate by antioxidants. Among the various factors contributing to NAFLD pathogenesis, oxidative stress has been considered the main contributor to liver injury and the progression of NAFLD. The strong effect of oxidative stress in the pathogenesis of liver diseases has been investigated in the last two decades^8^. Changing the diet is one of the most common preventive and therapeutic methods used in recent years to treat liver diseases, especially fatty liver^9^. It is known that antioxidant vitamins contribute to the body's defense against oxidative stress^10^. In this regard, a significant relationship between the consumption of fruits and vegetables rich in antioxidants and reducing the risk of fatty liver disease has been reported^11^. A recent systematic review and meta-analysis showed that a plant-based diet significantly reduced AST and GGT levels but had no significant effect on ALT^12^. On the other hand, contradictory results have been obtained regarding dietetics antioxidants and the levels of liver enzymes^13–17^.

Considering the importance of consuming foods containing antioxidants on liver health and the limited studies conducted in this regard on healthy Iranian adults, the purpose of this study was to evaluate the association between intake of some dietary antioxidants and levels of liver enzymes in a cross-sectional study based on the data of the Rafsanjan cohort study (RCS).

## Materials and methods

### Study design and participant selection

This cross-sectional study was conducted using the RCS data as a population-based prospective cohort, a part of the Prospective Epidemiological Research Studies in IrAN (PERSIAN)^18^. A total of 9991 subjects aged 35–70 years of both genders consented to participate in the enrollment phase of RCS that launched in August 2015 and ended in December 2017, and a 15-year follow-up was planned.

### Data collection

Through a standardized interview and validated questionnaire, data on socio-economic status, smoking habits, opium use, alcohol consumption, disease history, diet, and physical activity were collected. Moreover, height, weight, waist circumference, as well as systolic and diastolic blood pressure were measured by trained personnel based on the study protocol^19^. Socio-economic status was measured according to the wealth score index (WSI). This index was determined using multiple correspondence analysis (MCA) of the participants' variables, such as access to a washing machine, access to a computer, access to the internet, access to a car, owning a mobile phone, and international trips in a lifetime. Physical activity was measured according to the 24-h physical activity and a 22-item questionnaire. The information gathered in this questionnaire was converted to the metabolic equivalent of task hours per week [metabolic equivalent of task (MET)-h/week]. Cigarette smoking, alcohol drinking, and opium consumption were self-reported. Consumption of opium at least once per week for six months was defined as opium usage^20^. History of diseases including fatty liver, diabetes, hypertension, myocardial infarction (MI), and heart disease was recorded according to these questions "Do you have a history of fatty liver, diabetes, hypertension, or heart disease diagnosed by a physician?" The answer was yes or no. The main questionnaire used to collect the data was derived from the PERSIAN Cohort Web-based Electronic Standard Questionnaires^18^. The validity and reliability of questionnaires were assessed in previous studies^18,21–28^. Validity and reliability of the Food Frequency Questionnaire (FFQ) were evaluated in a prospective cohort study in northern Iran. To assess the validity, the results obtained from multiple FFQs with those from multiple 24-h diet recalls and biochemical markers of dietary intake in serum (b-carotene, retinol, vitamin C, and a-tocopherol) and urine (nitrogen) were compared. Moreover, to assess the reliability (reproducibility), intraclass correlation coefficients between results of four FFQs administered to the same participants were evaluated. The results showed good validity and reliability of the FFQ^21^.

### Dietary intake assessment

The participants completed a semi-quantitative FFQ with 118 items which asked about dietary intake over the past year by trained nutritionists. The participants were interviewed about each food item by two questions: (1) the number of times per month, week, or day the food was consumed during the previous year, and (2) the amount of the food that was usually consumed every time (portion size according to the standard serving sizes that Iranians usually consume). All reported intakes were converted to g/day using household portion sizes of consumed foods. Nutrient intakes were calculated using the United States Department of Agriculture (USDA) food database. In the present study, intakes of some antioxidants, including vitamin C, vitamin A, vitamin E, selenium, and carotenoids (α-carotene, β-carotene, lycopene, and β-cryptoxanthin) were assessed.

### Laboratory assessment

Blood samples were taken from all subjects between 7:00 and 9:00 a.m. after fasting for at least 12 h. Fasting blood sugar (FBS), total cholesterol, high-density lipoprotein cholesterol (HDL cholesterol), low-density lipoprotein cholesterol (LDL cholesterol), triglycerides (TG), AST, ALT, GGT, and ALP were measured by a biotecnica analyzer (BT 1500, Italy) at the Central Laboratory in Cohort center. Activities of serum ALT and AST were determined by kinetic method (Pars Azmoon Co, Tehran, Iran). In addition, as substrates, p-nitrophenol phosphate and p-Glutamyl-3-carboxy-4-nitroanilide phosphate were used for measuring serum ALP and GGT activities, respectively (kinetic method, Pars Azmoon Co, Tehran, Iran).

### Definition of terms

The laboratory's reference range in the Cohort center was used to define elevated ALT, AST, GGT, and ALP. Elevated ALT and AST were considered greater than 40 U/L and 35 U/L in males and females, respectively. Elevated GGT was defined as greater than 54 U/L and 37 U/L in males and females, respectively. Moreover, elevated ALP was defined as greater than 306 U/L for both genders.

### Statistical analyses

Baseline characteristics of individuals, including demographic characteristics, personal habits, and medical and laboratory characteristics, were compared across the groups of the present study (elevated liver enzymes) using chi-square (χ^2^) for categorical and t-test for continuous variables. Frequency (%) was used for categorical variables and the mean (SD: Standard Deviation) was employed for the quantitative variables. In addition, dichotomous logistics regression models were applied to investigate the association between elevated liver enzymes and intake of dietary antioxidants. The authors used two crude and adjusted models in the regression analysis and identified confounder variables using relevant epidemiological texts and according to subject matter knowledge. Potential confounding variables were sequentially entered into models according to their hypothesized strengths of association with liver enzyme levels and intake of dietary antioxidants. Then, variables with a p-value < 0.25 were selected as confounders. The crude model was stratified on the status of dietary antioxidants. In addition, the adjusted model was adjusted for confounding variables age (continuous variable), gender (male/female), education years (continuous variable), wealth status index, cigarette smoking (categorical), alcohol consumption (yes/no), opium consumption (yes/no), body mass index (BMI; continuous variable), physical activity level (continuous variable), diabetes (yes/no), hypertension (yes/no), MI (yes/no), heart disease (yes/no), triglycerides (continuous variable), LDL cholesterol (continuous variable), HDL cholesterol (continuous variable), using hepatotoxic drugs (yes/no), and fatty liver (yes/no). All analyses were performed through Stata V.14 (Stata Corp. 2015. Stata Statistical Software: Release 14. College Station, TX: Stata Corp LP), and all p-values are two-sided.

### Ethics approval and consent to participate

The ethics committee of Rafsanjan University of Medical Sciences approved this study (Ethical codes: ID: IR.RUMS.REC.1399.080). Written informed consent was obtained from the participants. The data of Participants kept confidential and was only accessible to the study investigators. All methods were performed in accordance with the relevant guidelines and regulations.

## Results

In the present study, 9942 participants from the baseline phase of the Rafsanjan adult cohort study, who had completed data on serum liver enzymes, were included. Among this population, 4631 (46.59%) were male, and 5311 (53.42%) were female. Table 1 presents the lifestyle variables, personal habits, socio-demographic characteristics, and anthropometric measures in individuals with normal and elevated liver enzymes. All elevated liver enzymes had significant associations with a higher mean BMI. Significant associations were shown between elevated ALT and ALP with age, BMI, education, WSI, alcohol consumption, cigarette smoking, and opium consumption. Moreover, there were significant associations between elevated ALT with gender and elevated ALP with physical activity. In addition, elevated AST was significantly associated with physical activity, BMI**,** alcohol consumption, and cigarette smoking, and elevated GGT was significantly associated with age, gender, education, physical activity, BMI, and WSI.

**Table 1:** The associations of the liver enzymes with demographic characteristics and personal habits in the study participants (n = 9942).

Characteristics	ALT		P -value	AST		P -value	GGT		P -value	ALP		P -value
	Normal	Elevated		Normal	Elevated		Normal	Elevated		Normal	Elevated	
Age cat- no. (%)			< 0.0001			0.61			< 0.0001			< 0.0001
Mean ± SD	50.21 ± 9.60	47.18 ± 8.84		49.95 ± 9.57	49.66 ± 9.49		49.80 ± 9.59	51.16 ± 9.25		49.64 ± 9.58	52.76 ± 8.97	
Gender- no. (%)			< 0.0001			0.621			0.001			0.827
Female	4963 (54.82)	348 (39.15)		5156 (53.46)	155 (52.01)		4716 (52.85)	595 (58.45)		4788 (53.38)	523 (53.75)	
Male	4090 (45.18)	541 (60.85)		4488 (46.54)	143 (47.99)		4207 (47.15)	423 (41.55)		4181 (46.62)	450 (46.25)	
Education-no. (%)			< 0.0001			0.063			< 0.0001			< 0.0001
Mean ± SD	8.45 ± 5.05	9.30 ± 5.04		8.55 ± 5.05	8.00 ± 5.14		8.61 ± 5.04	7.85 ± 5.12		8.69 ± 5.02	7.09 ± 5.10	
Physical activity- no. (%)			0.466			0.009			< 0.0001			0.01
Mean ± SD	38.79 ± 6.30	38.63 ± 6.81		38.80 ± 6.34	37.83 ± 6.48		38.88 ± 6.43	37.87 ± 5.48		38.83 ± 6.33	38.29 ± 6.54	
BMI- no. (%)			< 0.0001			< 0.0001			< 0.0001			< 0.046
Mean ± SD	27.70 ± 4.97	29.14 ± 4.27		27.78 ± 4.92	29.22 ± 4.91		27.67 ± 4.91	29.16 ± 4.83		27.79 ± 4.92	28.13 ± 5.00	
WSI-no. (%)			0.036			0.366			< 0.001			< 0.001
Mean ± SD	-0.005 ± 0.994	0.069 ± 1.058		0.003 ± 0.997	-0.050 ± 1.088		0.016 ± 0.01	-0.126 ± 0.031		0.028 ± 0.994	-0.243 ± 1.027	
Alcohol consumption- no. (%)			< 0.0001			0.006			0.945			0.045
Yes	844 (9.38)	142 (16.15)		943 (9.84)	43 (14.78)		885 (9.98)	101 (10.05)		872 (9.79)	114 (11.83)	
No	8150 (90.62)	737 (83.85)		8639 (90.16)	248 (85.22)		7982 (90.02)	904 (89.95)		8037 (90.21)	850 (88.17)	
Cigarette smoking -no. (%)			0.007			0.003			0.140			< 0.0001
Current	1549 (17.22)	120 (13.65)		1637 (17.08)	32 (11)		1514 (17.07)	155 (15.42)		1438 (16.14)	231 (23.96)	
Former	770 (8.56)	93 (10.58)		826 (8.620	37 (12.71)		786 (8.86)	77 (7.66)		777 (8.72)	86 (8.92)	
Never	6675 (74.22)	666 (75.77)		7119 (74.30)	222 (76.29)		6567 (74.06)	773 (76.92)		6694 (75.14)	647 (67.12)	
Opium consumption- no. (%)			0.001			0.227			0.583			< 0.0001
Yes	2160 (24.02)	168 (19.11)		2268 (23.67)	60 (20.62)		2098 (23.66)	230 (22.89)		2021 (22.68)	307 (31.85)	
No	6834 (75.98)	711 (80.89)		7314 (76.33)	231 (79.38)		6769 (76.34)	775 (77.11)		6888 (77.32)	657 (68.15)	

*AST* aspartate aminotransferase, *ALT* alanine aminotransferase, *ALP* alkaline phosphatase, *GGt* γ-glutamyl transferase, *BMI* body mass index, *FBS* Fasting blood sugar, *WSI* Wealth score index.

Table 2 indicates the associations of liver enzymes with the medical and laboratory characteristics of the study participants. The elevated level of four liver enzymes was significantly correlated with diabetes, triglyceride, LDL cholesterol, cholesterol, and FBS. Moreover, elevated serum levels of AST, GGT, and ALP were significantly associated with hypertension and MI. Elevated serum levels of ALT, GGT, and ALP were significantly associated with heart disease and HDL cholesterol. A significant association was observed between fatty liver with elevated ALT, GGT, and AST. Moreover, elevated serum levels of GGT and ALP were significantly associated with the consumption of hepatotoxic drugs.

**Table 2:** The associations of the liver enzymes with selected medical and laboratory characteristics in the study participants (n = 9942).

Variables	ALT		P -value	AST		P -value	GGT		P -value	ALP		P -value
	Normal	Elevated		Normal	Elevated		Normal	Elevated		Normal	Elevated	
Diabetes- no. (%)			0.041			0.013			< 0.0001			< 0.0001
Yes	1735 (19.25)	195 (22.11)		1856 (19.34)	74 (25.17)		1627 (18.32)	302 (29.90)		1649 (18.47)	281 (29.09)	
No	7276 (80.75)	687 (77.89)		7743 (80.66)	220 (74.83)		7255 (81.68)	708 (70.10)		7278 (81.53)	685 (70.91)	
Hypertension no (%)			0.391			0.019			< 0.0001			< 0.0001
Yes	2045 (22.69)	189 (21.43)		2151 (22.41)	83 (28.23)		1936 (21.80)	297 (29.41)		1917 (21.47)	317 (32.82)	
No	6966 (77.31)	693 (78.57)		7448 (77.59)	211 (71.77)		6946 (78.20)	713 (70.59)		7010 (78.53)	649 (67.18)	
Fatty liver- no (%)			< 0.001			< 0.0001			< 0.0001			0.409
Yes	843 (9.36)	167 (18.93)		957 (9.97)	53 (18.03)		845 (9.51)	165 (16.34)		904 (10.13)	106 (10.97)	
No	8168 (90.64)	715 (81.07)		8642 (90.03)	241 (81.97)		8037 (90.49)	845 (83.66)		8023 (89.87)	860 (89.03)	
MI- no. (%)			0.505			0.029			0.032			0.004
Yes	271 (3.01)	23 (2.61)		279 (2.91)	15 (5.10)		253 (2.85)	41 (4.06)		251 (2.81)	43 (4.45)	
No	8740 (96.99)	859 (97.39)		9320 (97.09)	279 (94.90)		8626 (97.150	969 (95.94)		8676 (97.19)	923 (95.55)	
Heart disease no (%)			0.008			0.267			0.003			0.007
Yes	809 (8.98)	56 (6.35)		834 (8.69)	31 (10.54)		751 (8.46)	114 (11.29)		758 (8.49)	107 (11.08)	
No	8202 (91.02)	826 (93.65)		8765 (91.31)	263 (89.490		8131 (91.54)	896 (88.71)		8169 (91.51)	859 (88.92)	
Using hepatotoxic drug- no (%)			0.207			0.254			< 0.0001			< 0.0001
Yes	2184 (24.24)	197 (22.34)		2302 (23.98)	79 (26.87)		2065 (23.25)	315 (31.19)		2077 (23.27)	304 (31.47)	
No	6827 (75.76)	685 (77.66)		7297 (76.02)	215 (73.13)		6817 (76.75)	695 (68.810		6850 (76.73)	662 (68.53)	
Triglyceride			< 0.0001			0.019			< 0.0001			< 0.0001
Mean ± SD	165.73 ± 106.72	203.62 ± 139.62		168.66 ± 111.15	183.92 ± 89.63		164.27 ± 105.22	211.67 ± 142.83		166.33 ± 103.57	194.85 ± 159.3	
LDL cholesterol			< 0.001			0.022			< 0.0001			0.007
Mean ± SD	107.84 ± 30.04	111.68 ± 32.76		108.06 ± 30.15	112.15 ± 34.93		107.61 ± 29.77	113.16 ± 34.34		107.91 ± 29.98	110.67 ± 33.14	
HDL cholesterol			< 0.0001			0.547			0.022			0.02
Mean ± SD	57.88 ± 10.90	56.44 ± 10.52		57.76 ± 10.84	57.38 ± 11.94		57.67 ± 10.82	58.49 ± 11.40		57.83 ± 10.78	57.00 ± 11.72	
Cholesterol			< 0.001			0.002			< 0.001			< 0.0001
Mean ± SD	197.88 ± 37.68	206.66 ± 41.02		198.46 ± 37.82	205.25 ± 45.02		197.19 ± 37.18	211.58 ± 43.04		204.22 ± 42.19	198.06 ± 37.55	
FBS			< 0.001			< 0.001			< 0.001			< 0.001
Mean ± SD	112.56 ± 38.26	120.56 ± 46.15		112.98 ± 0.39	123.07 ± 2.84		111.42 ± 0.39	129.52 ± 1.72		111.37 ± 35.49	130.91 ± 60.50	

*ALT* alanine aminotransferase, *AST* aspartate aminotransferase, *GGt* γ-glutamyl transferase, *ALP* alkaline phosphatase, *MI* Myocard Ischemia, *FBS* Fasting blood sugar.

Furthermore, Table 3 demonstrates the associations of liver enzymes with the mean dietary antioxidant intake. The results presented that elevated ALT had a significant association with the mean of Se, lycopene, Vit A, β-cryptoxanthin, and Vit E consumptions. A significant association was observed between subjects with elevated levels of GGT and the lower mean of Vit E consumption. In addition, the subjects with elevated levels of ALP had significantly lower mean of all antioxidant consumption except lycopene.

**Table 3:** The associations of the liver enzymes with the mean of dietary antioxidants in the study participants (n = 9942).

Variables	ALT		P -value	AST		P -value	GGT		P -value	ALP		P -value
	Normal	Elevated		Normal	Elevated		Normal	Elevated		Normal	Elevated	
Se (µg/day)			< 0.001			0.743			0.058			< 0.001
Mean ± SD	70.39 ± 46.75	77.72 ± 52.40		71.01 ± 47.43	71.93 ± 43.64		71.35 ± 47.70	68.37 ± 43.83		71.57 ± 47.34	66.23 ± 46.91	
β-Carotene (µg/day)			0.28			0.637			0.545			< 0.001
Mean ± SD	2995.14 ± 2487.82	3089.67 ± 2397.45		3001.51 ± 2482.25	3070.78 ± 2406.00		3008.74 ± 2504.39	2958.87 ± 2254.87		3031.38 ± 2496.39	2748.63 ± 2309.09	
α-Carotene (µg/day)			0.831			0.696			0.463			0.007
Mean ± SD	528.45 ± 749.92	534.06 ± 718.77		528.43 ± 749.32	545.71 ± 674.12		530.84 ± 756.43	512.60 ± 660.39		535.62 ± 754.31	467.81 ± 675.36	
Lycopenee (µg/day)			0.044			0.222			0.817			0.287
Mean ± SD	8588.09 ± 5651.29	8989.68 ± 5513.16		8611.81 ± 5633.67	9018.59 ± 5837.94		8628.22 ± 5658.95	8584.89 ± 5475.49		8643.88 ± 5607.76	8441.14 ± 5927.20	
Vit A (µg/day)			< 0.001			0.409			0.427			< 0.001
Mean ± SD	555.87 ± 328.61	596.10 ± 413.76		558.97 ± 337.38	575.44 ± 333.35		560.38 ± 333.47	551.50 ± 369.10		563.49 ± 338.42	522.52 ± 324.24	
Vit C (mg/day)			0.137			0.892			0.828			0.016
Mean ± SD	102.94 ± 71.11	106.64 ± 64.83		103.25 ± 70.78	103.82 ± 63.96		103.22 ± 70.77	103.73 ± 68.92		103.83 ± 70.13	98.08 ± 74.38	
β-Cryptoxanthin (µg/day)			0.027			0.513			0.583			0.001
Mean ± SD	212.44 ± 180.27	226.46 ± 177.93		213.48 ± 180.13	220.45 ± 179.39		214.00 ± 181.86	210.72 ± 163.80		215.62 ± 180.00	195.99 ± 180.23	
Vit E (mg/day)			0.001			0.152			0.013			< 0.001
Mean ± SD	5.47 ± 2.85	5.80 ± 2.78		5.49 ± 2.84	5.73 ± 2.80		5.52 ± 2.87	5.28 ± 3.00		5.53 ± 2.84	5.18 ± 2.80	

*AST* aspartate aminotransferase, *ALT* alanine aminotransferase, *ALP* alkaline phosphatase, *GGt* γ-glutamyl transferase, *Se* selenium, *µg/day* Micrograms per day, *mg/day* Milligrams per day.

Table 4 demonstrates the association of elevated liver enzymes with dietary antioxidant intake using the crude and adjusted models. According to this table, in the crude model, higher odds of elevated ALT were significantly associated with higher intake of Se, β-carotene, lycopene, Vit A, Vit C, β-cryptoxanthin, and Vit E; however, this positive association was not observed after adjusting for all confounder variables. Moreover, in the crude model, lower odds of elevated GGT were significantly associated with higher intake of Se, Vit A, and Vit E, and this association was not seen in the fully adjusted model. The decreased odds of elevated ALP had a significant association with the higher intake of Se, β-carotene, α- carotene, lycopene, Vit A, Vit C, β-cryptoxanthin, and Vit E in the crude model, which remained significant even in the adjusted model except for lycopene and Vit C. In the fully adjusted model, in subjects with higher consumption of Se, β-carotene, α-carotene, Vit A, and β-cryptoxanthin, the odds ratios of elevated ALP were decreased in the 4th quartile compared to 1st quartile (ORs: 0.79 (0.64–0.96), 0.79 (0.64–0.96), 0.78 (0.64–0.95), 0.80 (0.66–0.98), 0.79 (0.64–0.98) respectively). In the fully adjusted model, the odds ratio of elevated ALP in subjects with consumption of Vit E in the 3rd quartile was 0.73 (0.60–0.89), which was significantly lower than that of the subjects in the 1st quartile. Moreover, after multivariate logistic regression, we graphed the predicted probabilities of elevated ALP against the quartiles of Se, Vit A, Vit E, β-carotene, α-carotene, β-cryptoxanthin intakes using the command *margins* in STATA (Fig. S1).

**Table 4:** Association of the dietary antioxidants and odds of elevated liver enzymes in study participants using the crude and adjusted models.

Antioxidants	Elevated ALT		Elevated AST		Elevated GGT		Elevated ALP	
	Crude OR	Adjusted OR	Crude OR	Adjusted OR	Crude OR	Adjusted OR	Crude OR	Adjusted OR
Se (µg/day)								
Quartile 1	1	1	1	1	1	1	1	1
Quartile 2	1.20 (0.97–1.47)	1.01 (0.81–1.26)	0.89 (0.63–1.25)	0.88 (0.62–1.26)	0.86 (0.72–1.03)	0.95 (0.78–1.14)	0.76 (0.63–0.91)	0.87 (0.72–1.05)
Quartile 3	1.26 (1.02–1.55)	0.93 (0.75–1.16)	1.12 (0.81–1.55)	1.17 (0.83–1.65)	0.78 (0.65–0.93)	0.88)0.72–1.07)	0.68 (0.57–0.82)	0.83 (0.68–1.01)
Quartile 4	1.62 (1.33–1.97)	1.03 (0.83–1.29)	1.10 (0.79–1.52)	1.01 (0.71–1.44)	0.85 (0.71–1.02)	0.90 (0.74–1.10)	0.67 (0.56–0.81)	0.79 (0.64–0.96)
β-Carotene** (**µg/day)								
Quartile 1	1	1	1	1	1	1	1	1
Quartile 2	1.22 (1.00–1.48)	1.07 (0.87–1.31)	1.17 (0.85–1.61)	1.21 (0.87–1.68)	1.03 (0.86–1.23)	1.06 (0.87–1.28)	0.86 (0.72–1.02)	0.94 (0.78–1.13)
Quartile 3	0.99 (0.80–1.21)	0.82 (0.66–1.02)	0.75 (0.53–1.07)	0.75 (0.52–1.10)	0.97 (0.81–1.17)	1.01 (0.83–1.23)	0.71 (0.59–0.85)	0.81 (0.67–0.99)
Quartile 4	1.18 (0.97–1.44)	0.94 (0.75–1.16)	1.13 (0.82–1.55)	1.15 (0.81–1.62)	1.00 (0.83–1.20)	1.04 (0.85–1.27)	0.67 (0.55–0.80)	0.79 (0.64–0.96)
α-Carotene** (**µg/day)								
Quartile 1	1	1	1	1	1	1	1	1
Quartile 2	1.16 (0.95–1.42)	1.10 (0.89–1.35)	1.18 (0.85–1.64)	1.18 (0.84–1.66)	1.06 (0.88–1.27)	1.05 (0.87–1.27)	0.84 (0.70–1.00)	0.89 (0.74–1.07)
Quartile 3	1.15 (0.94–1.40)	1.13 (0.92–1.40)	1.04 (0.74–1.47)	1.11 (0.78–1.58)	0.95 (0.79–1.14)	0.97 (0.80–1.18)	0.77 (0.64–0.92)	0.84 (0.70–1.02)
Quartile 4	1.08 (0.89–1.32)	0.97 (0.78–1.20)	1.18 (0.85–1.64)	1.21 (0.86–1.72)	0.98 (0.82–1.18)	0.99 (0.81–1.20)	0.69 (0.57–0.83)	0.78 (0.64–0.95)
Lycopene** (**µg/day)								
Quartile 1	1	1	1	1	1	1	1	1
Quartile 2	0.98 (0.80–1.20)	0.91 (0.74–1.13)	1.03 (0.73–1.45)	1.08 (0.76–1.53)	0.90 (0.75–1.09)	0.96 (0.79–1.16)	0.89 (0.74–1.07)	0.97 (0.80–1.17)
Quartile 3	1.31 (1.08–1.59)	1.11 (0.91–1.37)	1.25 (0.90–1.73)	1.24 (0.88–1.75)	0.94 (0.78–1.13)	0.99 (0.82–1.20)	0.91 (0.76–1.10)	1.06 (0.88–1.29)
Quartile 4	1.16 (0.95–1.42)	0.93 (0.76–1.15)	1.20 (0.86–1.67)	1.22 (0.86–1.72)	0.93 (0.77–1.12)	0.98 (0.81–1.19)	0.85 (0.71–1.03)	0.95 (0.79–1.16)
Vit A** (**µg/day)								
Quartile 1	1	1	1	1	1	1	1	1
Quartile 2	1.29 (1.05–1.58)	1.10 (0.88–1.35)	0.93 (0.67–1.30)	0.97 (0.69–1.36)	0.90 (0.75–1.08)	0.97 (0.80–1.17)	0.72 (0.60–0.87)	0.83 (0.70–1.01)
Quartile 3	1.20 (0.98–1.48)	0.92 (0.74–1.14)	0.89 (0.64–1.24)	0.90 (0.63–1.28)	0.77 (0.64–0.93)	0.85 (0.70–1.04)	0.70 (0.59–0.84)	0.86 (0.71–1.04)
Quartile 4	1.39 (1.14–1.70)	0.96 (0.77–1.20)	1.11 (0.81–1.52)	1.12 (0.79–1.58)	0.93 (0.78–1.11)	1.02 (0.84–1.24)	0.66 (0.55–0.80)	0.80 (0.66–0.98)
Vit C** (**mg/day)								
Quartile 1	1	1	1	1	1	1	1	1
Quartile 2	1.06 (0.86–1.30)	0.98 (0.79–1.21)	0.82 (0.58–1.15)	0.86 (0.61–1.23)	1.05 (0.88–1.27)	1.15 (0.95–1.40)	0.91 (0.76–1.09)	1.03 (0.86–1.24)
Quartile 3	1.36 (1.12–1.66)	1.16 (0.94–1.44)	1.10 (0.80–1.51)	1.16 (0.83–1.63)	0.99 (0.82–1.20)	1.10 (0.90–1.35)	0.82 (0.68–0.98)	0.99 (0.81–1.20)
Quartile 4	1.21 (0.99–1.48)	0.92 (0.73–1.15)	1.01 (0.73–1.40)	1.02 (0.71–1.45)	1.01 (0.89–1.28)	1.19 (0.97–1.46)	0.69 (0.57–0.83)	0.85 (0.69–1.05)
β-Cryptoxanthin** (**µg/day)								
Quartile 1	1	1	1	1	1	1	1	1
Quartile 2	1.31 (1.07–1.60)	1.21 (0.98–1.50)	0.93 (0.67–1.29)	0.96 (0.68–1.34)	0.99 (0.82–1.19)	1.07 (0.89–1.30)	0.93 (0.78–1.10)	1.07 (0.89–1.28)
Quartile 3	1.30 (1.06–1.59)	1.11 (0.89–1.38)	0.92 (0.66–1.28)	0.90 (0.63–1.28)	0.97 (0.81–1.17)	1.06 (0.87–1.30)	0.76 (0.63–0.91)	0.90 (0.74–1.10)
Quartile 4	1.41 (1.15–1.72)	1.09 (0.87–1.37)	1.02 (0.74–1.41)	1.02 (0.71–1.46)	1.02 (0.85–1.22)	1.14 (0.93–1.40)	0.63 (0.52–0.76)	0.79 (0.64–0.98)
Vit E** (**mg/day)								
Quartile 1	1	1	1	1	1	1	1	1
Quartile 2	1.01 (0.82–1.24)	0.86 (0.69–1.07)	0.91 (0.65–1.28)	0.93 (0.65–1.31)	0.80 (0.67–0.96)	0.85 (0.71–1.03)	0.76 (0.64–0.91)	0.88 (0.73–1.06)
Quartile 3	1.22 (1.00–1.48)	0.96 (0.77–1.19)	0.87 (0.62–1.23)	0.91 (0.63–1.30)	0.83 (0.70–0.99)	0.94 (0.77–1.14)	0.58 (0.48–0.70)	0.73 (0.60–0.89)
Quartile 4	1.36 (1.12–1.65)	0.88 (0.71–1.10)	1.31 (0.96–1.80)	1.33 (0.93–1.88)	0.76 (0.63–0.91)	0.83 (0.68–1.02)	0.70 (0.58–0.84)	0.90 (0.73–1.10)

The crude model is stratified on the status of antioxidant intake.

The adjusted model is adjusted for confounding variables age (continuous variable), gender (male/ female), education years (continuous variable) ,wealth status index, variables related to lifestyle: cigarette smoking (categorical), alcohol drinking (yes/no), and opium consumption (yes/no), body mass index (continuous variable), physical activity level (continuous variable), diabetes (yes/no), hypertension (yes/no), MI (yes/no), heart disease (yes/no), triglycerides (continuous variable), LDL cholesterol (continuous variable), HDL cholesterol (continuous variable), using hepatotoxic drugs (yes/no) and fatty liver (yes/no).

*ALT* alanine aminotransferase, *AST* aspartate aminotransferase, *GGt* γ-glutamyl transferase, *ALP* alkaline phosphatase, *MI* Myocardial Ischemia, *Se* Selenium, *µg/day* Micrograms per day, *mg/day* Milligrams per day.

In addition to adjusting for using hepatotoxic drugs and fatty liver, we performed a sensitivity analysis since some of the increased odds of elevated liver enzymes probably are driven from residual confounding due to using hepatotoxic drugs or fatty liver. After excluding the subjects that used hepatotoxic drugs or had fatty liver, no association was observed between dietary intake of antioxidants and the levels of serum liver enzymes.

## Discussion

In the present study, we assessed the association of some dietary antioxidant intakes, including vitamin A, vitamin C, vitamin E, selenium, and carotenoids (α-carotene, β-carotene, lycopene, and β-cryptoxanthin) with odds of elevated liver enzymes in a large population of Iranian adults. In people with higher consumption of Se, Vit A, Vit E, and provitamin A carotenoids (β-carotene, α-carotene, β-cryptoxanthin), the odds ratios of elevated ALP were decreased compared to the reference group. These findings support the hypothesis that Se, Vit A, Vit E, and provitamin A carotenoids may be associated with improvements in ALP and act as suppressors against the development of liver injury. However, after performing a sensitivity analysis, no association was observed between dietary intake of antioxidants and the levels of serum liver enzymes, which could be due to the residual confounding effect caused by fatty liver or consuming hepatotoxic drugs. It is also important to mention that fatty liver can probably act as an intermediate variable in relation to the intake of antioxidants and the increase in liver enzymes so that the reduction of the intake of antioxidants can increase the risk of fatty liver and, subsequently, the fatty liver increases the level of liver enzymes.

There is limited evidence regarding the association between dietary antioxidant intakes and odds of elevated liver enzymes among healthy Iranian adults. Previous results have demonstrated that increased dietary intake^13,15,29,30^ of antioxidants was related to lower levels of serum liver enzymes. In line with the results of the present investigation, a previous cross-sectional study on 5111 Iranian adults aged 35–70 years old demonstrated that the consumption of phytochemicals was associated with improvements in ALP. There was an inverse association between the dietary phytochemical index (DPI) score (the percent of daily energy intake taken from phytochemical-rich foods) and serum ALP in the adjusted model. No significant associations were found between DPI score and elevated serum levels of AST, ALT, and GGT^13^.

Similarly, in a cross-sectional study on 1791 Japanese employees aged 18–69 years, the association of dietary non-enzymatic antioxidant capacity (NEAC) in overall diet with serum AST, ALT, and GGT levels was evaluated. After adjustment for confounding factors, no significant associations were found between overall dietary NEAC intake and the levels of these liver enzymes^15^. Moreover, in a cross-sectional study including 7960 apparently healthy Japanese men aged 22–86 years, green tea consumption as a polyphenol antioxidants source was not associated with serum GGT^17^.

In contrast with our results, Abazarfard et al. showed lower ALT and AST levels in women with an almond-enriched diet as a good source of fiber and antioxidants^31^. A recent systematic review and meta-analysis of randomized controlled trials found that the Mediterranean diet, as a plant-based diet, significantly reduced AST and GGT but had no significant effect on ALT^12^.

Previous results have shown that higher blood concentrations of antioxidants were inversely associated with serum liver enzymes^14^. In a longitudinal study of 1073 participants aged 30–79 years at the baseline phase of the Mikkabi cohort study, after adjusting for confounders, reduced risks for elevated serum ALT were significant for serum β-carotene, β-cryptoxanthin and total provitamin A carotenoids over a mean follow-up period of 7.8 years. Moreover, reduced risks were not significant for α-carotene, non-provitamin A carotenoids, and lycopene^14^.

Furthermore, intakes of fruits and vegetables, as a rich source of antioxidants, were correlated with lower levels of liver enzymes in some studies^16,29,32^; however, the results of another investigation were not in line with this claim^30^. In a cross-sectional study conducted on 265 healthy adults with a mean age of 35 who were living in Tehran city in Iran, individuals in the upper quartile of vegetable intake were less likely to have elevated ALT and AST levels. After controlling for potential confounders, only the association between vegetable intake and ALT level remained significant. Liver enzymes had no significant relationship with the quartiles of fruit intake^16^. In addition, Nanri et al. reported an inverse relationship between serum GGT levels and a diet rich in fruits and vegetables^32^.

Some previous studies evaluated the association between antioxidant micronutrients and NAFLD^33–35^. In a cross-sectional analysis of 72 patients diagnosed with NAFLD, the association between serum and dietary antioxidants and liver fibrosis was evaluated. The NAFLD-diagnosed patients showed a significant serum deficiency of retinol (20.8%), vitamin C (27%), and selenium (73.6%), as well as insufficient dietary intake of vitamin A (98.3%) and vitamin E (100%). Patients with advanced liver fibrosis had reduced levels of serum retinol^33^. In a case–control study in Iran, with 196 NAFLD patients and 803 people in the control group, the association between polyphenol consumption during the year prior to the investigation and the risk of NAFLD was evaluated. Accordingly, higher total polyphenol intake was associated with a lower risk of NAFLD^34^. Moreover, Ekhlasi et al. observed that ALT and AST levels decreased and total antioxidant capacity increased in NAFLD patients who consumed pomegranate juice as an antioxidant and polyphenol-rich source^35^.

The heterogeneity in the results of the present study compared with other studies might be due to differences in sample size, genetic background, geographically various antioxidant content of foods, the method used to assess dietary intake, study design, the health status of participants, different level of adjustment for confounders, and analysis method.

The large sample size, population-based research, and extensive information regarding potential confounders, especially those of liver injury (e.g., fatty liver disease and consumption of hepatotoxic drugs), were the main strengths of the present research. Another major strength of the current investigation was the measurement of four enzymes (ALT, AST, GGT, and ALP) for liver function assessment. Moreover, in the present study, face-to-face interviews were used to ask about FFQ data, making these data more reliable. In addition, another positive point of this study was that the anthropometric measurements were conducted instead of self-reporting. Despite of these advantages, there were also several limitations. First, since the type of the present study was cross-sectional, a causal association could not be inferred. Accordingly, it is suggested that this relationship be reconsidered in the follow-up phase of this prospective study. Second, FFQ is susceptible to recall bias; the subjects might under- or overestimate their food consumption during the year before the investigation. Another limitation was that we could not measure oxidative stress parameters to evaluate their influence on the activity of liver enzymes. Finally, alcohol use was included in the final model as a categorical variable (yes/no). Given that alcohol use^36^ has a dose-dependent effect on elevated liver enzymes, the probability of residual confounding effect could be in our results.

## Conclusion

Subjects with higher consumption of Se, Vit A, Vit E, and provitamin A carotenoids (β-carotene, α-carotene, β-cryptoxanthin) showed decreased odds of elevated ALP. These findings support the hypothesis that Se, Vit A, Vit E, and provitamin A carotenoids may be associated with improvements in ALP and act as suppressors against the development of liver injury.

## Supplementary Information

Supplementary Figure S1.

## Data Availability

The datasets used during the current study are available on the Persian Adult Cohort Study Center, Rafsanjan University of Medical Sciences, Iran. The data is not available publicly. However, upon a reasonable request, the data can be obtained from the corresponding author.
