# Supplementary material for: Dietary antioxidants and liver enzymes in Rafsanjan, a Region in Southeast Iran
Source: Sci Rep. 2023 May 26;13:8555. doi: 10.1038/s41598-023-35385-0 (PMC10220050; doi:10.1038/s41598-023-35385-0)
Supplement: Supplementary file 1 — Supplementary Figure S1. [file 41598_2023_35385_MOESM1_ESM.docx]

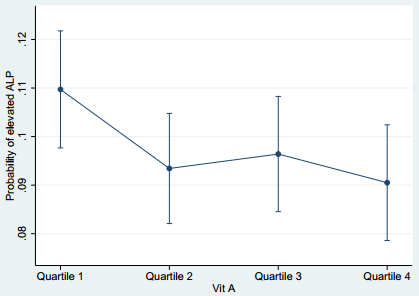

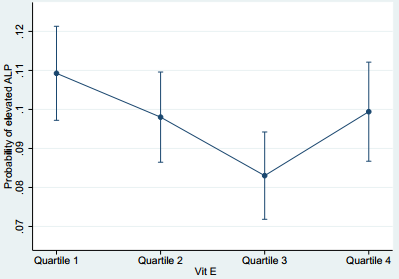

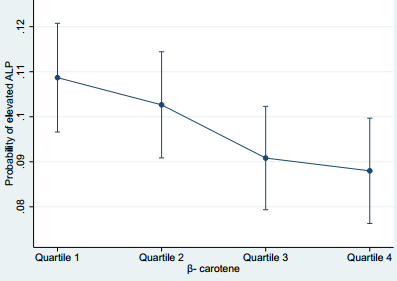


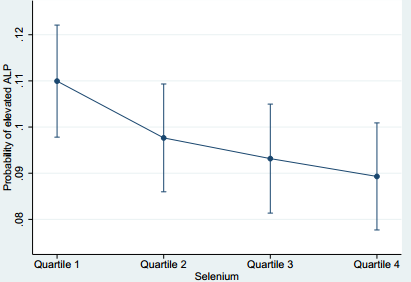

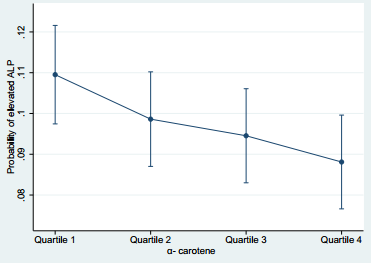

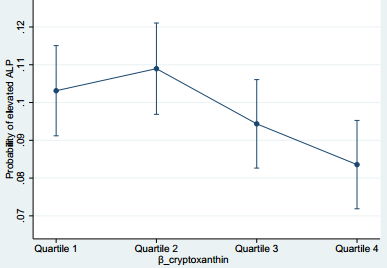


Figure S1. The predicted probabilities of elevated ALP against the categories of dietary Se, Vit A, Vit E, β-carotene, α-carotene, β-cryptoxanthin intakes.
